# Environmental gut bacteria in European honey bees (*Apis mellifera*) from Australia and their relationship to the chalkbrood disease

**DOI:** 10.1371/journal.pone.0238252

**Published:** 2020-08-28

**Authors:** Sheba Khan, Doug Somerville, Michael Frese, Murali Nayudu

**Affiliations:** 1 Faculty of Health, University of Canberra, Canberra, Australia; 2 NSW Department of Primary Industries, Goulburn, NSW, Australia; 3 Faculty of Science and Technology, University of Canberra, Canberra, Australia; Universitat Leipzig, GERMANY

## Abstract

We report on aerobic “environmental” bacteria isolated from European honey bees (*Apis mellifera*). We determined the number of culturable aerobic bacteria in the gut of nurse bees sampled from locations around Australia. Bees from healthy colonies had 10^7^–10^8^ aerobic bacteria per g of bee gut, while bees from colonies with chalkbrood consistently had significantly fewer bacteria (10^4^–10^5^ bacteria per g). When colonies recovered from chalkbrood, bacterial numbers returned to normal levels, suggesting that counting aerobic bacteria in the gut could be used to predict an outbreak of the disease. Furthermore, Western Australian bees from the “Better Bees” program (bred to promote hygienic behaviour) had significantly higher numbers of aerobic gut bacteria compared to regular bees from healthy colonies. Bacteria with the ability to inhibit the chalkbrood pathogen were found in most bees from regular colonies (> 60%) but only in a few “Better Bees” (10%). Phylogenetic analysis of aerobic bacterial isolates that inhibited the chalkbrood pathogen revealed a close relationship (>97% sequence identity) to the genera *Bacillus*, *Klebsiella*, *Pantoea*, *Hafnia*, and *Enterobacter* (bacteria that have previously been isolated from honey bees), but we also isolated *Maccrococcus* and *Frigoribacterium* species (bacteria that were not previously identified in bees). Finally, we investigated the ability of bacteria to inhibit the chalkbrood fungus *Ascosphaera apis*. Mass spectroscopy analysis revealed that the bee gut isolates *Frigoribacterium* sp. and *Bacillus senegalensis* produce gluconic acid. We further found that this simple sugar is involved in chalkbrood fungal hyphal lysis and cytoplasmic leakage. Our findings suggest that “environmental” gut bacteria may help bees to control the chalkbrood pathogen.

## Introduction

European honey bees (*Apis mellifera*) were first introduced to Australia in 1822 for honey production and soon became widespread. Bees are not only key pollinators of many agricultural crops but also became important pollinators of some native Australian plant species [1]. The production of honey and honey-derived products directly contributes 101 million AUD per annum to the Australian economy. More importantly, honey bees provide pollination to a wide range of agricultural and horticultural crops with an estimated benefit to the economy of about 2 billion AUD per annum [2]. In Australia, European honey bees have very different pollen and nectar sources, compared with other continents [3]. The unique flora that has evolved on the Australian “island” continent since the breakup of the Gondwana supercontinent in the Jurassic period [4] enables the production of some unique honeys, such as Leatherwood honey and *Leptospermum* (Manuka) honey [5]. Furthermore, Australia is still free of the *Varroa* mite [6] and colony collapse disorder (CCD)—in contrast to other parts of the world [7].

Chalkbrood is a disease of the honey bee brood that is caused by the heterothallic fungus *Ascosphaera apis* [8]. In Australia, the chalkbrood disease was first detected in 1993; it quickly became endemic [9] and is now the most important disease in the local bee industry [10]. Physical, chemical, and biological stress factors predispose colonies to chalkbrood, such as extreme (high and low) temperatures, high humidity, environmental pollution, pesticide poisoning, parasite infestations, and predator attacks [11]. The severity of a chalkbrood outbreak depends on the pathogenicity of the fungus, the vitality of the colony (e.g., hygienic behavior), and specific environmental factors [12]. Some measures can be taken to reduce the probability of disease outbreaks, such as maintaining colonies with sufficient numbers of bees, not allowing bees to winter in brood chambers that are too large [13], or re-queening the colony with a queen from a more resistant stock [14]. Enlarging hive entrances to aid ventilation and reduce moisture accumulation may also help [13]. In a chalkbrood-infested colony, each infected larva can produce up to 10^8^–10^9^ spores [8]. Chalkbrood mummies are frequently ejected from the colony by nurse bees, which reduces the pathogen load in the hive. Thus, the hygienic behavior of nurse bees is a key factor in halting the spread of the disease and in promoting recovery [15].

A wide range of microorganisms were found to be associated with honey bees and their food (i.e., nectar, pollen, honey, beebread, and propolis [16]). The gut microbiome of bees and other insects has been studied in great detail. For example, microorganisms digest and ferment plant cell wall components [17], produce essential vitamins [18], and prevent harmful pathogens from colonizing the gut through the occupation of key niches [18] and/or the production of metabolites that prime and/or enhance host immune responses [17]. Furthermore, it has been postulated that bee gut bacteria contribute to general hygiene, pathogen inhibition, and bee bread preservation [19, 20]. Molecular analysis of honey bee gut microbial communities revealed eight characteristic, strictly anaerobic or microaerophilic phylotypes that were found across several environments and geographical locations [21–24]. The most frequently found “core” bacteria of adult honey bees are Gram-negative Proteobacteria (e.g., *Snodgrassella* and *Gilliamella* ssp. [25]) and Gram-positive Firmicutes (e.g., *Lactobacillus* and *Lactobacillus* ssp. [26]). Less frequently found are *Bifidobacterium* ssp. [27], *Frischella*, *Bartonella*, *Parasaccharibacter*, and *Gluconobacter*-related species [26]. Bees acquire these bacteria early in life and most retain them throughout their lifetime [28]. Martha Gilliam first showed that the bee gut also contains a diverse range of aerobic bacterial species and that some of these bacteria (e.g., *Bacillus* ssp.) inhibit the chalkbrood pathogen [20]. These “environmental” bacteria may only colonize the bee gut opportunistically but nevertheles may have important roles to play in the digestion of food or the control of pathogens.

This study reports on aerobic bacteria from the gut of European honey bees from Australia. We chose to investigate nurse bees, because they rarely leave the hive, look after the brood, and are responsible for feeding the larvae, which means that it is the gut bacteria of nurse bees that are most likely passed on to the next generation of bees [29, 30]. Furthermore, nurse bees remove infected chalkbrood larvae from the hive [15]. Thus, nurse bees are key players in both the spread and the recovery from chalkbrood [15, 29, 30]. By comparing nurse bees from healthy colonies with those from chalkbrood-infected colonies in the same apiaries, we tested the hypothesis that changes to the number of culturable (aerobic) gut bacteria correlate with the appearance (or disappearance) of the chalkbrood disease. Furthermore, using 16S DNA sequencing, we determined the identity of selected aerobic bee gut bacteria that were found to inhibit the fungal pathogen *Ascosphaera apis*, and we investigated the mechanism by which bacteria inhibit the growth of *A*. *apis*.

## Materials and methods

The main steps involved in the isolation and characterization of aerobic gut bacteria from Australian honey bees are summarized in a flowchart (S1 Fig).

### Honey bee sampling and processing of the bee gut

Samples were collected from the major honey producing regions across Australia during the Australian late spring, summer, and early autumn (when bees are most active). We sampled from the Australian Capital Territory (ACT; 7 hives), New South Wales (NSW; 29 hives), Queensland (QLD; 10 hives), Victoria (VIC; 10 hives), Tasmania (TAS; 10 hives), South Australia (SA; 6 hives), and Western Australia (WA; 20 hives). Each hive was deconstructed for sampling, assessed for the presence of chalkbrood, and bees taken from the brood area, which made it highly likely that most sampled bees were indeed nurse bees. Most bees were transported to the laboratory on the same day where they were kept at room temperature until they were killed for the isolation of aerobic bacteria from the bee gut. If bees were shipped by mail (over-night), they were sent in queen mailing cages plugged with irradiated sugar candy.

For each honey bee colony, bacteria from four randomly selected nurse bees were analyzed. Bees were placed in a fridge at 4°C for a minimum of three but not more than four hours. The bees were carefully removed from the fridge, the sternum of each bee was crushed using sterile tweezers, and a second pair of sterile tweezers was used to pull out the entire gastrointestinal tract (i.e., ventriculus, ileum, and rectum). The crop was cut off, the remaining gut was transferred into an Eppendorf tube with 1 ml of sterile dilute nutrient broth (Sigma-Aldrich; catalogue number, 03856), and the fresh weight was determined (to three decimal places).

### Quantification of bacteria

The bee gut was crushed using sterile sticks and vortexed for 1 min. Serial dilutions of the resulting suspension were prepared in nutrient broth and 200 μl of each dilution along with the neat (both in duplicates) was spread over the surface of agar plates with a sterile spreader. The plates were incubated at 25°C for 2 days. Bacterial counts were determined on tryptic soy agar (TSA [31]) on dilution plates that had countable numbers of bacteria (i.e., 20–200 colonies per plate). We calculated the number of bacteria per g bee rather than per bee to allow meaningful comparisons of bee gut bacterial numbers across colonies and apiaries from different environments/geographical locations.

All bacterial counts (CFU/g) were log_10_-transformed and evaluated statistically using Genstat, version 9.0 [32]. The Mann-Whitney U (Wilcoxon rank-sum) test for non-parametric analysis was used to compare different samples. Observed differences were considered significant at *P* < 0.05. In conjunction with the Mann-Whitney U test, a one-way analysis of variance (ANOVA) was carried out to compare the means and the least significant difference between sample groups.

### Isolation of bacteria

Bacteria were isolated on TSA (Oxoid; catalogue number, CM0131), a general purpose medium; eosin methylene blue agar (EMB; Oxoid; catalogue number, CM0069) for enteric bacteria; modified Gould medium (mS1) for the selective isolation of *Pseudomonas* sp.; and glucose-calcium carbonate medium (G-CaCO_3_) for the selective isolation of *Gluconobacter* ssp. [31]. Potato dextrose agar (PDA; Oxoid; catalogue number, CMO139) was used for fungus inhibition assays and the isolation of bacterial hydrophilic compounds. For light microscopy studies, thinly plated PDA plates were poured that contained a lower potato dextrose concentration than normal (i.e., 10 g/liter). The pH indicator bromocresol purple (5,5’-dibromo-o-cresolsulfonphthalein) was added to PDA to detect the production of acidic bacterial metabolites. The indicator was added to the medium (15 mg/liter) before autoclaving and the pH was adjusted through the dropwise addition of 5 M NaOH until the medium was deep purple in color (pH 6.8); a color change to yellow (pH < 5) indicated acidification. Bacteria were streaked on media and incubated at 25°C for 2 days. Potato dextrose broth (PDB; Fisher Scientific; catalogue number, BD 254920) was used to grow bacteria for preparing crude extracts of the *Pseudomonas* strain AN5 and the AN5 transposon mutant derivatives AN5MN1 and AN5MN2. *Pseudomonas* strain AN5 produces gluconic acid (approximately 0.5 mg/ml) on PDA medium, while the two independent transposon mutants AN5MN1 and AN5MN2 do not produce any gluconic acid [33].

### Gram staining

Bacteria were stained using a Gram staining kit (Sigma-Fluka; catalogue number 77730) and observed under a light microscope (at a magnification of 1,000×).

### Chalkbrood inhibition assay

All bacterial isolates were tested for their ability to inhibit the chalkbrood and/or the “take-all” fungus using previously described standard bioassays [20]. Briefly, plugs of chalkbrood were placed between the middle and the rim of PDA plates before the plates were streaked with bacteria on the opposite side. Control plates had the fungal plug placed in the middle of the plate. The plates were then incubated for 10–14 days at 18°C and fungal inhibition was determined with an Alpha Digidoc image system (Sigma Aldrich) that quantifies the plate surface area with fungal growth. Results are expressed as mean % inhibition, using the following formula [34]: % inhibition = [1- (fungal growth/control growth)] × 100.

### DNA isolation and sequencing

Total genomic DNA was isolated from selected bacterial strains using the DNeasy Blood & Tissue Kit (Qiagen; catalogue number, 69504). Multiplex PCR was used to amplify 16S rRNA gene fragments using the Multiplex PCR Kit (Qiagen; catalogue number, 206143). 16S rRNA amplification was carried out with the two primers: primer BSF 8/20 (5’-AGAGTTTGATCCTGGCTCAG-3’) and primer BSR 534/18 (5’-ATTACCGCGGCTGCTGGC-3’). These primers were designed using sequence information from the European Ribosomal RNA website [35]. Agarose gel electrophoresis was used to visualize the amplified 500-bp 16S rRNA DNA fragment. Bands with the correct size were excised from gels and purified using the Qiagen QIA quick Gel Extraction Kit. DNA sequencing of this fragment was performed at the Australian Genome Research Facility (AGRF) at the University of Queensland, Brisbane.

Partial 16S rRNA gene sequences were corrected and trimmed (as needed) using Sequencer 6.0 before they were subjected to a BLAST search of the GenBank non-redundant database to identify 16S rRNA gene sequences with greater than 96% identity and the highest level of similarity, as estimated using expect values [36]. For sequence comparisons, we used only those 16S rRNA gene sequence in the NCBI data base that were derived from known characterized bacterial strains. Where possible, sequences from strains of the American Type Culture Collection (ATCC) were used for creating the phylogenetic tree. Partial 16S rRNA gene sequences were determined for 19 bacterial isolates (Table 1).

**Table 1:** Classification, name and origin of aerobic bacteria isolated from the gut of Australian honey (nurse) bees.

Cluster	Bacterial isolate	Origin of isolate
*Bacillus* group	16H10Pw4.9	Bermagui (NSW)
	16H13Tw3.12	Bermagui (NSW)
	17H2Gw4.15	Launceston (TAS)
	17H3Gw3.4	Launceston (TAS)
	21H1Gw3.10	Perth (WA)
	23H1Tw4.20	Cooke Plains (SA)
	24H1Tw3.19	Cooke Plains (SA)
	30H1Tw2.10	Tubbut (VIC)
	30H2Gw2.10	Tubbut (VIC)
	30H2Gw2.12	Tubbut (VIC)
	30H2Gw3.18	Tubbut (VIC)
	30H2Gw3.5	Tubbut (VIC)
	ET6	Canberra (ACT)
Enteric group	14H12Pw3.4	Goulburn (NSW)
	16H13Pw2.4	Bermagui (NSW)
	16H14Ew3.3	Bermagui (NSW)
	20H2Ew2.1	Perth (WA)
*Macrococcus* group	ET2	Canberra (ACT)
*Frigoribacterium* group	DT4	Canberra (ACT)

16S rDNA sequences from bacterial isolates and known GenBank sequences were grouped using similarity scores and knowledge of their taxonomy. Separate datasets were compiled for each major grouping. Out group sequences from other bacterial genera, including a representative from each of the groups identified in this work, were included in each sequence dataset. Sequence datasets were aligned using the Fast Fourier Transform (MAFFT) alignment program [37], a program that is frequently used to align large numbers of sequences for phylogenetic analysis [38]. Maximum likelihood phylogenetic trees were generated by using PhyML 3.0 and sub-tree pruning and regrafting from 10 random starting trees [39]. Each of the substitution models available in PhyML were used, including the most complex model, a general time-reversible model with a proportion of invariant sites, and a gamma distribution of site-rate variants across categories: GTR+I+G [40]. Bootstrap values > 50% are shown at the nodal branches. Corresponding phylogenetic trees were derived from the partial 16S rRNA gene sequences.

### Anti-fungal compound extraction and detection

The aerobic ET2 bee gut bacterial isolate was spread onto five PDA plates and incubated at 25°C for five days before the contents of the plates were cut into cubes and transferred to a 250-ml flask with 100 ml of a water/isopropanol solution (40:60). The flask was plugged with bungs and placed on a shaker for 1 h, before the contents were strained and centrifuged at 6,000 rpm for 15 min. The pellet (agar) was discarded and the supernatant was freeze-dried, resuspended in 100 ml of acetone, transferred to a new glass beaker, and refrigerated at 4°C overnight. After another centrifugation step (6,000 rpm for 10 min), the sample was transferred to a new glass beaker and left uncovered in a laminar flow hood until all of the acetone had evaporated [33]. Finally, the sample was resuspended in 5 ml of sterile water. All isolations were performed in duplicate.

A 1-ml aliquot of the ET2 hydrophilic extract was tested for the presence of gluconic acid in a VG QUATTRO II mass spectrometer [33]. Mass spectrometry of pure gluconic acid (Merck; catalogue number, Sigma-Aldrich G1951) was carried out as a control. Methanol was used between samples to purge compound traces from the mass spectrometer. A collision-induced dissociation (CID) analysis of the putative gluconic acid peak was also performed to confirm the presence of gluconic acid polymers on any gluconic acid peaks observed.

Crude extracts of 2-day PDB cultures of the *Pseudomonas* strain AN5, or that of the independent transposon mutants AN5MN1 and AN5MN2, were obtained by filtration. The filtrate was then freeze-dried to concentrate any metabolites and resuspended in sterile water for use in bioassays [33]. All tests were performed as duplicates. Briefly, a plug of the chalkbrood fungus was placed in the middle of a thinly poured agar plate, sterile paper discs were placed symmetrically in each quadrant, and 100 μl of either water (as control) or crude bacterial extracts were pipetted onto each disc. The plates were then incubated at 18°C for 8 days and the effect of the various treatments were examined by direct observation under a light microscope (at a magnification of 400×).

## Results

### Nurse bees from healthy colonies have more aerobic gut bacteria than nurse bees from chalkbrood-infected colonies

**Fig 1:**
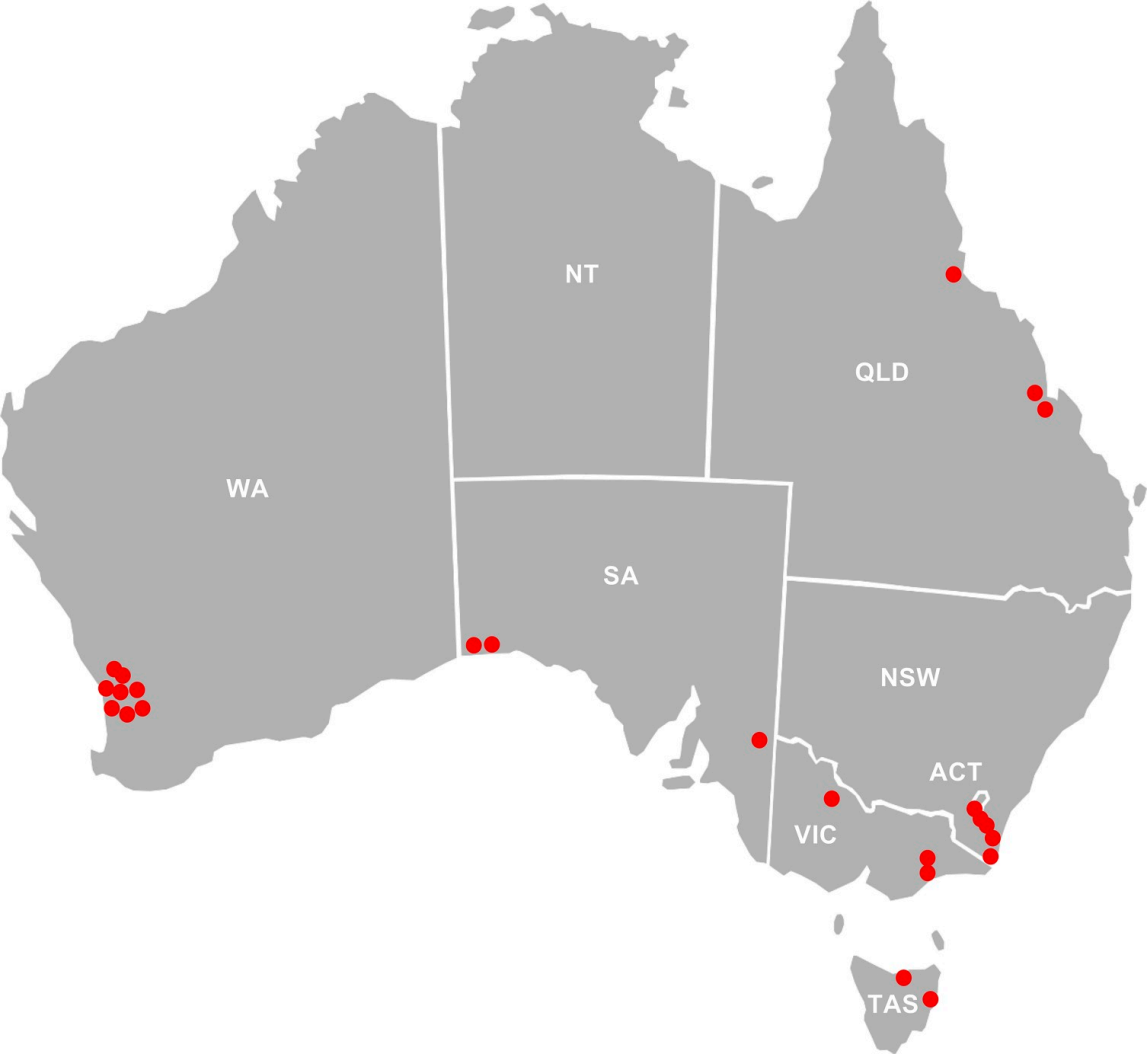
Origin of samples. Australian states and territories from which nurse bees were collected for this study. Red dots indicate the location of apiaries sampled in this study. NSW, New South Wales; VIC, Victoria; WA, Western Australia; QLD, Queensland; SA, South Australia; TAS, Tasmania; ACT, Australian Capital Territory.

For this study, 98 bee colonies were sampled from 33 different locations across Australia, covering the major honey production areas in all states and territories except the Northern Territory (Fig 1). When chalkbrood-infected colonies were sampled, healthy colonies from the same apiary were also sampled. From each colony that was selected for sampling, nurse bees were removed from the brood area, taken to the laboratory, and processed for the isolation of bee gut bacteria. Only the mid- to hindgut was harvested for bacterial isolation. The weight of the bee gut varied considerably (i.e., between 10 and 50 mg). Therefore, we calculated the number of bacteria per gram of bee gut to better compare bee gut bacterial numbers across different colonies and apiaries. Of note, we did not attempt to cultivate the anaerobic or microaerophilic “core” bacteria of the bee gut, our methods were chosen to analyze the abundance of aerobic “environmental” gut bacteria.

**Fig 2:**
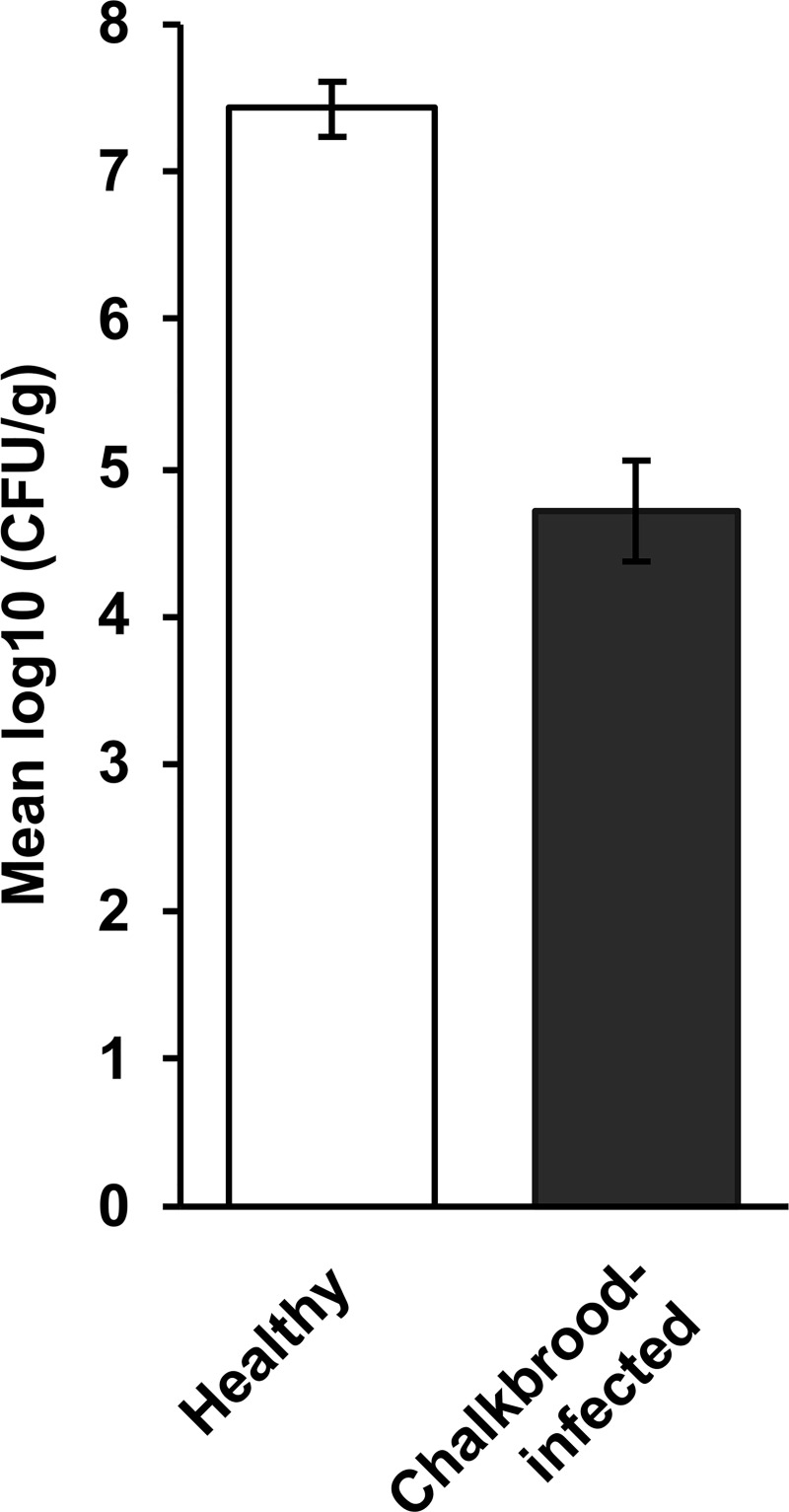
Aerobic gut bacterial numbers (CFU/g) in nurse bees from healthy hives (*n* = 47) and chalkbrood-infected hives (*n* = 31). Bees were collected from around Australia. The difference between the two groups is statistically significant (*P* < 0.001). Error bars represent the standard error of the mean (SEM) with a 5% least significant difference (LSD) level of 0.716.

All bees from healthy colonies (47 hives, 4 bees per hive, i.e., *n* = 47) had high numbers of aerobic gut bacteria (10^7^–10^8^ CFU/g) compared with bees from chalkbrood-infected colonies (31 hives, 4 bees per hive, i.e., *n* = 31), which had significantly (*P* < 0.001) fewer bacteria (10^4^–10^5^ CFU/g; Fig 2).

**Fig 3:**
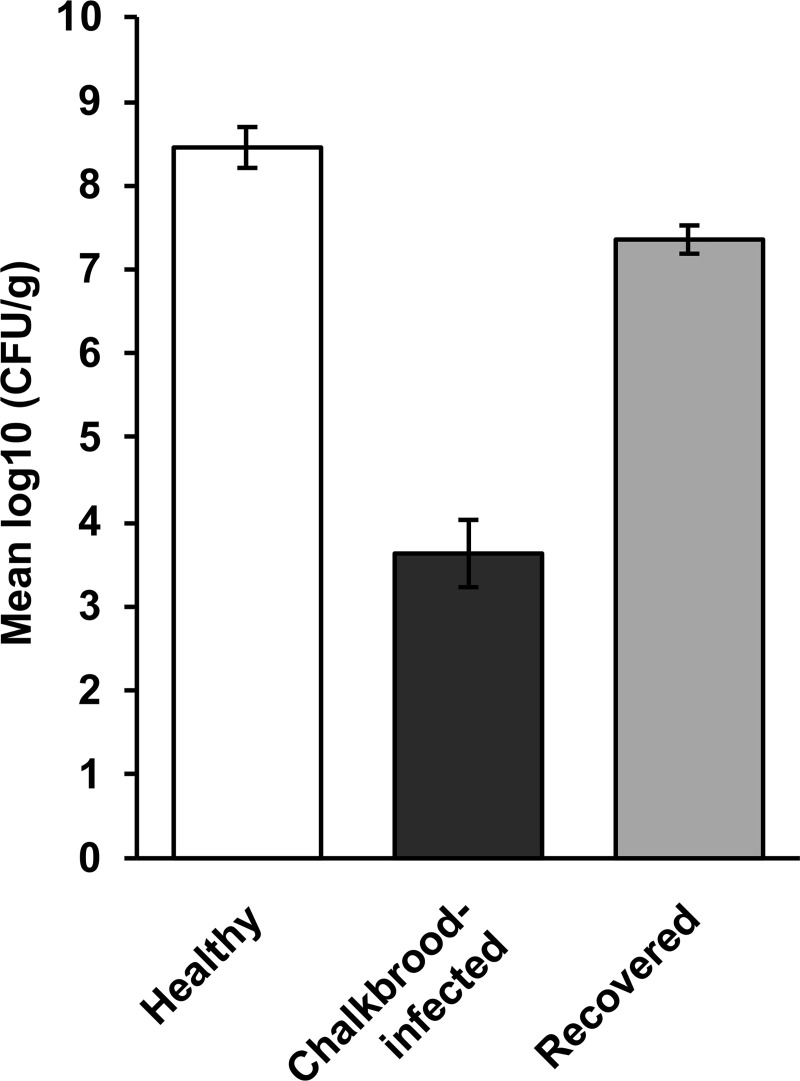
Aerobic gut bacterial numbers (CFU/g) in nurse bees from healthy hives (*n* = 10), chalkbrood-infected hives (n = 10), and chalkbrood-infected hives 21 weeks after the disappearance of disease signs (recovered; *n* = 10). All bees were collected from a single apiary in New South Wales. The differences between all groups are statistically significant (*P* < 0.001). Error bars represent the SEM with a 5% least significant difference (LSD) level of 1.095.

We also investigated changes to the number of aerobic gut bacteria in chalkbrood-infected hives over time in an apiary near the township of Goulburn (New South Wales) that had numerous chalkbrood-infected hives. From that apiary, bees from 8 colonies were sampled when the colonies showed signs of the chalkbrood disease. The same hives were sampled again 21 weeks later when the colonies had recovered, and the signs of the disease were no longer observed. For comparisons, bees from healthy colonies in the same apiary were also sampled (Fig 3). The results confirm our earlier findings that bees from chalkbrood-infected colonies contained fewer bacteria than bees from colonies that did not display any signs of the disease. Interestingly, the disappearance of the chalkbrood disease largely coincided with a recovery in bacterial numbers, even if aerobic bacterial numbers in the gut of bees from colonies that had just recovered from chalkbrood were slightly lower compared with bees from healthy colonies without a (recent) history of chalkbrood.

**Fig 4:**
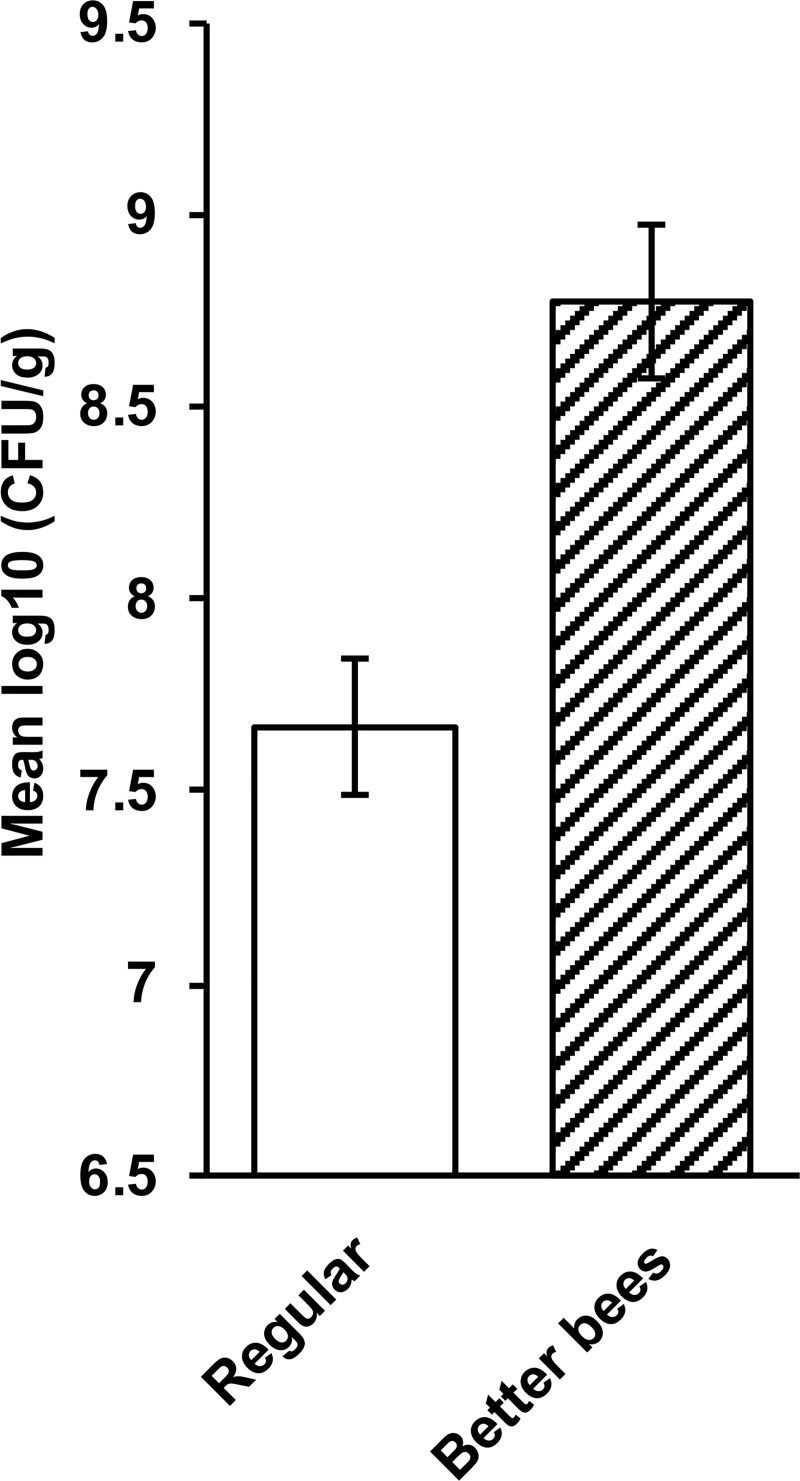
Aerobic gut bacterial numbers (CFU/g) in healthy nurse bees from “Better Bee” colonies (*n* = 10) and bees from regular breeding colonies (*n* = 10). All bees were collected from apiaries in Western Australia. The difference between the two groups is statistically significant (*P* = 0.002). Error bars represent the SEM with a 5% least significant difference (LSD) level of 0.562.

The results inspired us to analyze bees from the Western Australian “Better Bees” program. The “Better Bees” were bred for enhanced activity and improved hygienic behavior [41]. We compared aerobic bacterial bee gut counts from 10 healthy “Better Bee” colonies in Western Australia with normal bee colonies from the same area. The results show that bees from the “Better Bees” program had significantly more aerobic gut bacteria than bees from regular bee colonies (i.e.,10^8.5^–10^9^ bacteria vs 10^7^–10^8^ bacteria per g, respectively; *P* = 0.002; Fig 4).

### Bees from “Better Bee” colonies have fewer aerobic chalkbrood inhibiting gut bacteria than normal colonies

Of all healthy colonies that were tested, 61% had bees containing aerobic gut bacteria that inhibited the chalkbrood pathogen. Interestingly, 73% of colonies tested with chalkbrood symptoms had bees with aerobic gut bacteria that inhibited the chalkboard pathogen. The percentage increased even further to 83% for colonies that had recently recovered from chalkbrood. Somewhat unexpectedly, only 10% of the “Better Bee” colonies had bees with chalkbrood-inhibiting bacteria, which is significantly below that of other apiaries from Western Australia. This suggests that the “resistance” to chalkbrood in the “Better Bee” lines is not based on the presence of chalkbrood-inhibiting bacteria. There was no obvious correlation between the geographical location of an apiary and the number of chalkbrood-inhibiting gut bacteria. However, more apiaries need to be tested to confirm this result.

### Characterization of aerobic bee gut bacteria that inhibit the chalkboard pathogen

**Fig 5:**
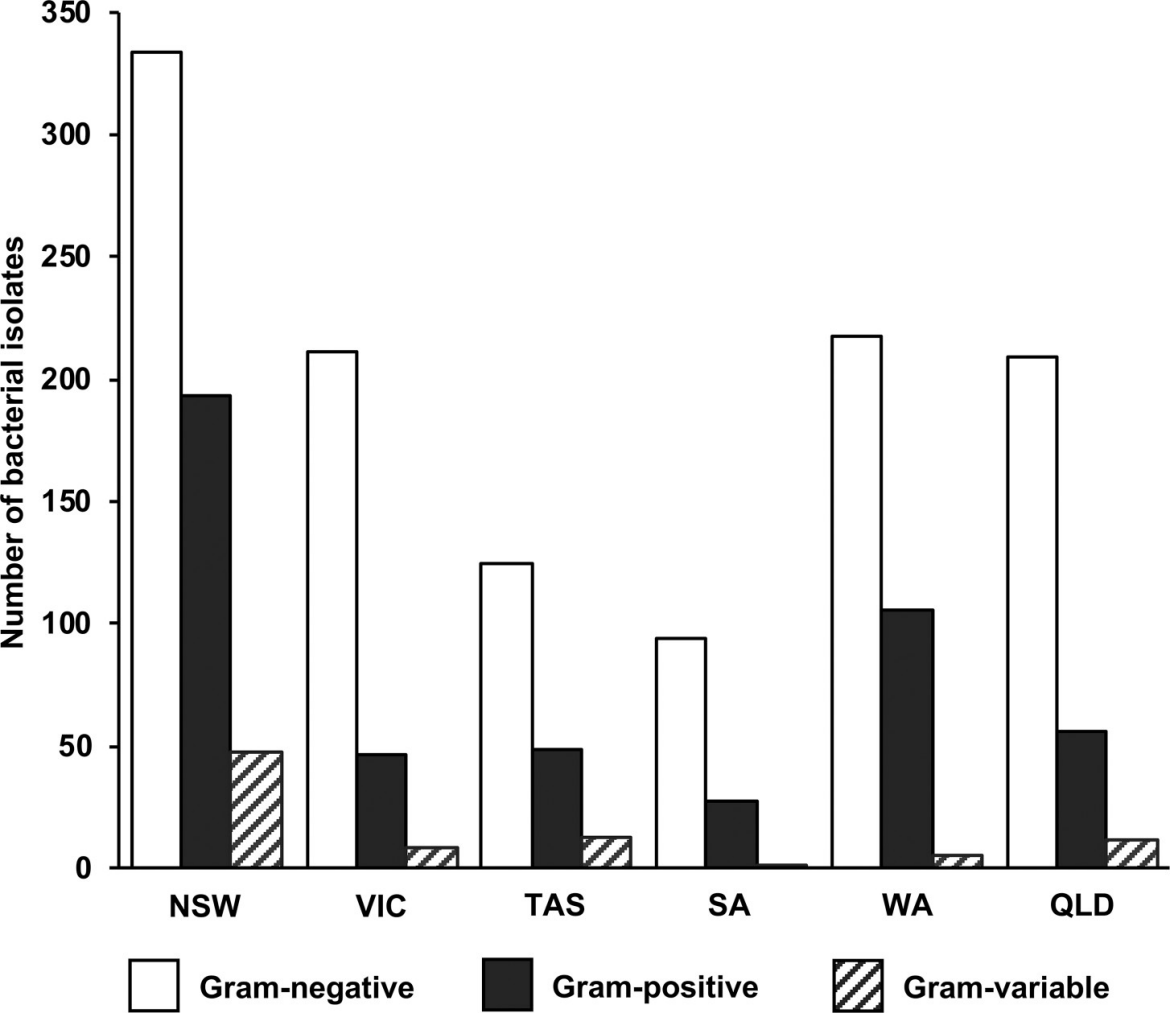
Gram staining properties of aerobic gut bacteria isolated from European nurse honey bees from in Australia. Isolates were analyzed from New South Wales (NSW), Victoria (VIC), Tasmania (TAS), South Australia (SA), Western Australia (WA), and Queensland (QLD).

To determine bee gut bacterial numbers, bacteria were usually grown on TSA media, but we also inoculated EMB, mS1 Gould, and glucose-CaCO_3_ agar to isolate bacteria that do not grow readily on TSA agar (S2 Fig). Gram staining was carried out for all bacterial isolates obtained in this study. We found that, throughout Australia, more Gram-negative than Gram-positive bacteria were observed in the gut of healthy bees (Fig 5), which is in line with earlier observations by Gilliam [20]. We also isolated a small number of Gram-variable bacteria (Fig 5).

**Fig 6:**
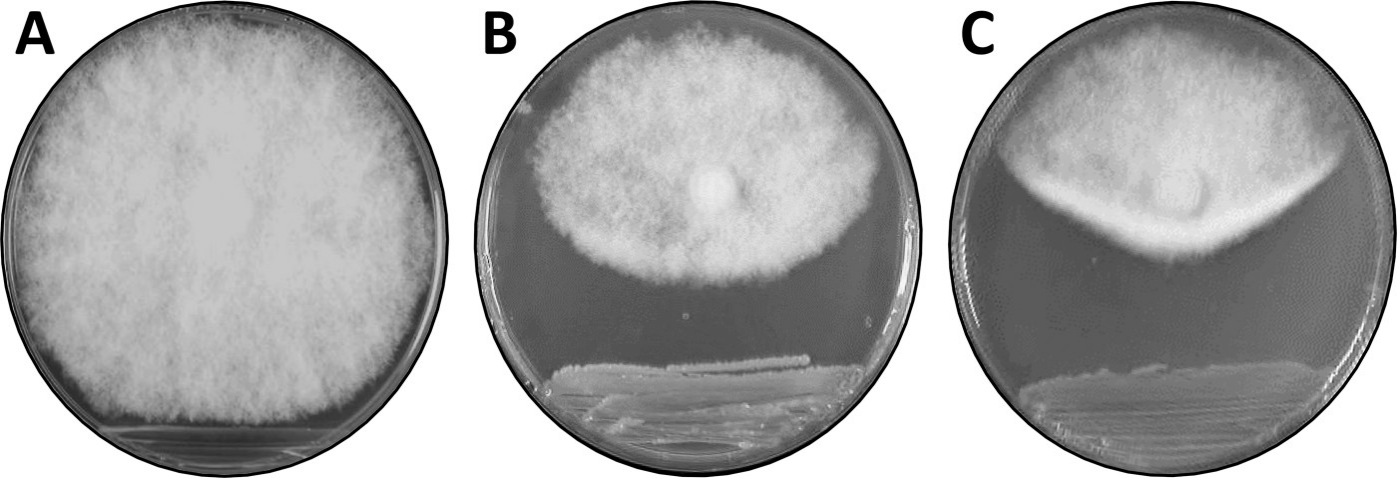
Chalkbrood bioassays. A chalkbrood fungal plug was placed at the top end of the plate with bacteria streaked at the bottom and incubated at 18°C for 10 days. (**A**) bacterial bee gut isolate 23H1Gw4.8 (no inhibition), (**B**) bacterial bee gut isolate 28H4Tw4.20 (inhibition, > 30%), and (**C**) *Pseudomonas* strain AN5 (inhibition, > 30%).

Of the 1,758 aerobic bacterial isolates from the gut of healthy bees, only 170 strongly inhibited the chalkbrood fungus (% inhibition, ≥ 30%; for examples without and with anti-fungal activity, see Fig 6A and 6B, respectively). A genetic analysis using randomly amplified polymorphic DNA revealed that 158 of the 170 isolates are not closely related. Next, we investigated the 158 aerobic bee gut isolates that were different from each other and that strongly inhibited the chalkbrood pathogen. The nature of metabolites produced by selected bacterial isolates was determined using PDA plates supplemented with the pH indicator bromocresol purple. Results indicate that aerobic bee gut bacteria produced a range of acidic to alkaline metabolites (as exemplified in S3 Fig).

### Gluconic acid production by bacteria and its effect on the chalkbrood pathogen

By growing bacteria on PDA plates, we found that one of our chalkbrood-inhibiting aerobic bacterial bee gut isolates, ET2, produced acidic metabolites when glucose, galactose, or mannose were provided as the sole carbon source (S3 Fig). This metabolic activity is indicative of the presence of a glucose oxidase-like enzyme [42]. The production of gluconic acid is of interest, because this simple sugar is involved in the inhibition of the “take-all” wheat pathogen *Gaeumannomyces graminis* var. *tritici* (Ggt) [33]. Thus, it was not surprising to find that ET2 also strongly inhibited the “take-all” pathogen in a PDA plate bioassay (% inhibition, ≥ 30%; similar to the inhibition of chalkbrood by the *Pseudomonas* strain AN5; Fig 6C). This observation suggests that ET2 produces gluconic acid, and that gluconic acid contributes to the inhibition of the chalkbrood pathogen. As gluconic acid is a water-soluble compound, we extracted hydrophilic metabolites from ET2-inoculated PDA plates and analyzed the extracts using mass spectroscopy. The analysis revealed a peak at approximately 195 m/z that is characteristic of gluconic acid. Collision-induced dissociation (CID) analysis of this 195-m/z peak revealed gluconic acid polymers (S4 Fig), confirming that the peak indeed represents gluconic acid [43]. Using similar methods, we found that the aerobic bee gut isolates DT4 and ET6 also produce gluconic acid but at a lower level than what was observed for ET2 (i.e., 20 and 10% lower, respectively).

In previous studies, we developed *Pseudomonas* strain AN5 as an effective biocontrol agent for the wheat “take-all” pathogen. We further showed that this bacterial strain produces gluconic acid and that this metabolite is essential for the suppression of the “take-all” pathogen [33]. Here, we report that strain AN5 also strongly inhibits the chalkbrood pathogen (% inhibition, ≥ 30%; Fig 6C) in contrast to two independent transposon mutants of AN5, AN5MN1 and AN5MN2 [33] that did not produce gluconic acid and were unable to inhibit the chalkbrood pathogen (i.e., in our bioassay, the chalkbrood fungus grew over the colonies of these AN5 mutants).

**Fig 7:**
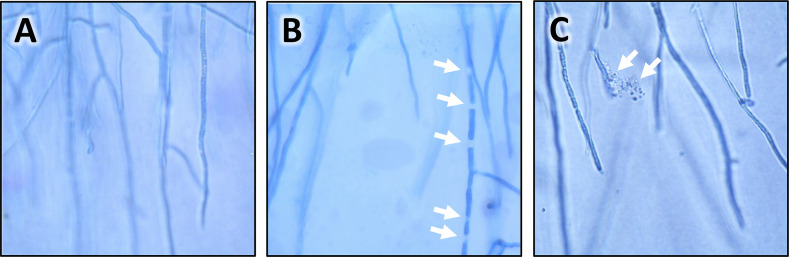
Bacterial metabolite-mediated cytopathic effects on hyphae of the chalkbrood fungus. Light microscopy images of chalkbrood mycelia that were grown on thin potato dextrose agar (PDA). (**A**) Mycelium of an untreated control; (**B**, **C**) mycelia that were treated with a semi-purified crude extract of *Pseudomonas* strain AN5. In panel B, the arrows indicate fragmentation due to a breakdown of septae; in panel C, the arrows indicate hyphal lysis and cytoplasmic leakage. Magnification, 1,000×.

Subsequent experiments, designed to further investigate the effect of gluconic acid on the chalkbrood pathogen, were conducted with *Pseudomonas* strain AN5 rather than the aerobic bee gut isolate ET2, as AN5 produces more gluconic acid than ET2. The switch to strain *Pseudomonas* AN5 allowed us to take advantage of the two AN5 transposon mutants that do not produce gluconic acid [33]. To investigate how *Pseudomonas* strain AN5 inhibits chalkbrood, semi-purified crude bacterial extracts were made from the wild-type strain and the two mutants, AN5MN1 and AN5MN2. While extracts from the wild-type AN5 strain strongly inhibited the chalkboard fungus, extracts from the mutant strains showed no inhibition. This result confirmed our hypothesis that gluconic acid production is essential for the observed inhibition of the chalkbrood pathogen. Some of the chalkbrood inhibition bioassays were performed on thin PDA plates, so light microscopic studies of the chalkbrood mycelia could be carried out. After 8 days of incubation at 18°C, normal fungal growth is characterized by mycelia that consist of filaments that branch often and have regular septae (Fig 7A). When a semi-crude extract of the *Pseudomonas* strain AN5 was added to a paper disc near the fungal plug, we observed a breakdown of the septae, hyphal lysis, and cytoplasmic leakage (Fig 7B and 7C), while neither the extracts of the two transposon mutants AN5MN1 and AN5MN2, nor the addition of water did affect the growth of the chalkbrood mycelia.

### Genetic characterization of aerobic bee gut bacteria that inhibit the chalkbrood pathogen

**Fig 8:**
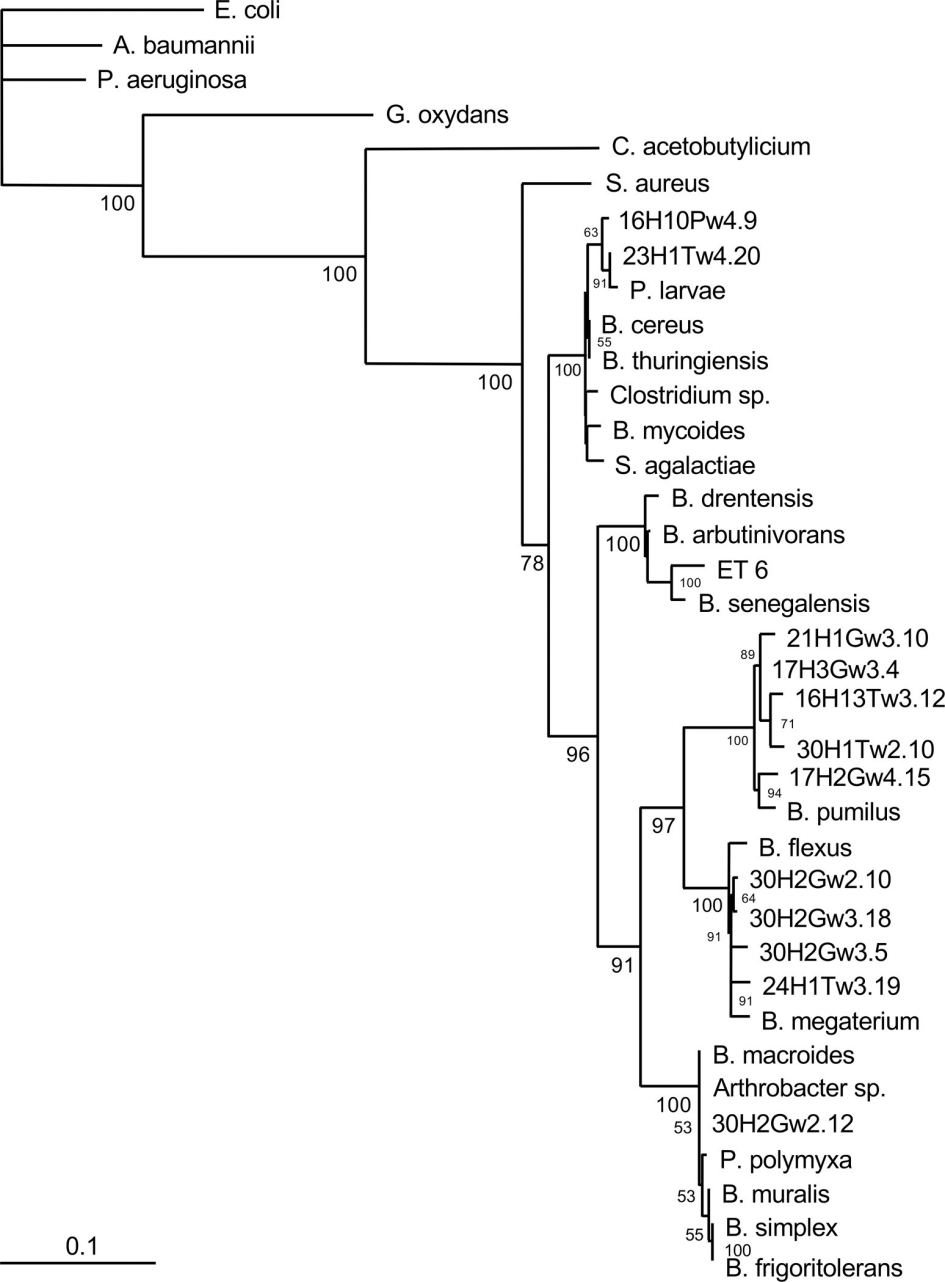
Phylogenetic analysis of 16S rRNA sequences (cluster I; *Bacillus* strains). Complementary DNA was amplified from aerobic bacteria isolated from the gut of nurse bees (*Apis millifera*) from Australia, sequenced, and sequences were aligned using the Fast Fourier Transform (MAFFT). A maximum likelihood phylogenetic tree was generated using PhyML3.0 and the general time reversible (GTR) evolutionary model. Bootstrap values detected for 100 replicates are shown near the nodes. The scale bar represents the change in nucleotides of the sequence (i.e., genetic variation for the length of scale).

**Fig 9:**
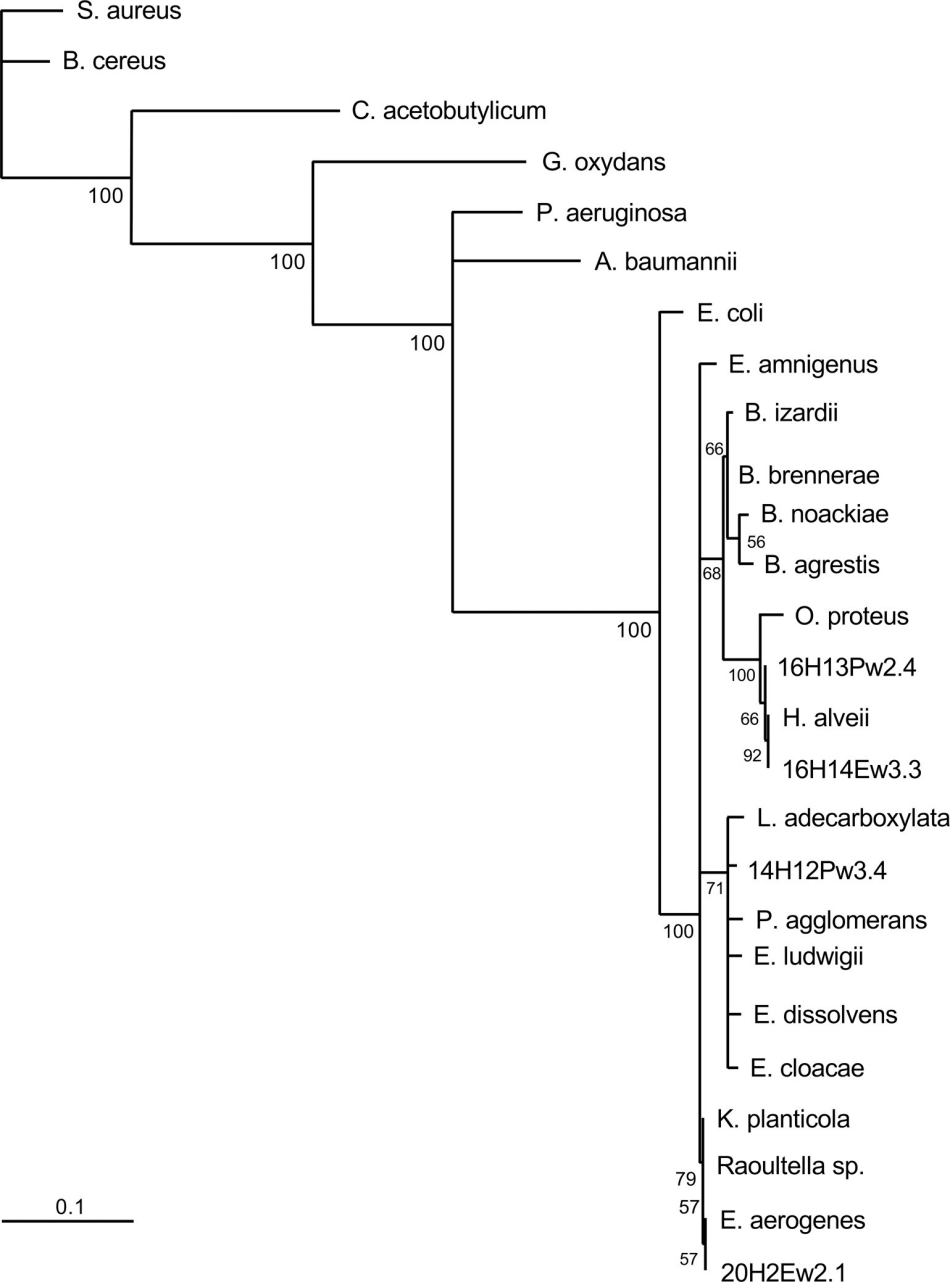
Phylogenetic analysis of 16S rRNA sequences (cluster II; enteric bacteria). Complementary DNA was amplified from aerobic bacteria isolated from the gut of nurse bees (*Apis millifera*) from Australia, sequenced, and sequences were aligned using the Fast Fourier Transform (MAFFT). A maximum likelihood phylogenetic tree was generated using PhyML3.0 and the general time reversible (GTR) evolutionary model. Bootstrap values detected for 100 replicates are shown near the nodes. The scale bar represents the change in nucleotides of the sequence (i.e., genetic variation for the length of scale).

Nineteen aerobic bee gut bacterial isolates (Table 1) that strongly inhibited the chalkboard pathogen, were chosen for identification. We chose these 19 strains to represent different geographic locations, Gram staining characteristics, and other properties of interest (e.g., gluconic acid production). In each case, a 500-bp region of the 16S rRNA gene was amplified by PCR with universal primers, Sanger sequenced, and an NCBI Blast search was carried out. The 16S rRNA gene sequence of the aerobic bacterial bee gut isolate 16H10Pw4.9 was found to perfectly match that of *Bacillus thuringiensis* strain bias Z2 (sequence similarity, 100%; E value = 0). Sequences of the other 18 aerobic bee gut bacterial isolates were analyzed in a similar manner and allowed us to define the following four clusters (sequence similarities > 97%): cluster I, *Bacillus* strains; cluster II, enteric strains; cluster III, *Macrococcus* strains; and cluster IV, *Frigoribacterium* and *Actinobacterium* strains. The phylogenetic relationship of the Gram-positive *Bacillus* isolates of cluster I (13 isolates) is shown as a phylogenetic tree (Fig 8). In some cases, however, analyzing 16S rRNA sequences was not sufficient to determine the phylogenetic relationships between very closely related species. For example, the aerobic bee gut isolate 16H10Pw4.9 showed 100% sequence identity to *Bacillus thuringiensis* strain bios Z2, *Bacillus mycoides* strain BGSC 6A13, and *Bacillus cereus* strain JSCtot9-2; further sequence analyses using other gene sequences (e.g., gyrase sequences) is required to identify the closest relative of this isolate. Cluster II contains four isolates with sequence similarities to Gram-negative enteric bacteria. Two isolates, 16H13Pw2.4 and 16H14Ew3.3, showed a high degree of sequence similarity to *Hafnia alvei* (> 97%; E value = 0). Isolate 14H12Pw3.4 showed sequence similarity to the *Enterobacter* species *Leclercia adecarboxylata* (99%; E value = 0), and the sequence of isolate 20H2Ew2.1 was identical to an *Enterobacter aerogenes* sequence (100%; E value = 0). The phylogenetic tree of cluster II is shown in Fig 9. Similar phylogenetic analyses were carried out with clusters III and IV. The only cluster III isolate, ET2, was identical to *Macrococcus hajekii* (100%; E value = 0), and the only cluster IV isolate, DT4, was identical to *Frigoribacterium* species (100%; E value = 0). Bootstrap values of > 50% for all branches (of all trees) indicate the robustness of the analyses and gives strong validity to the phylogenetic relationships observed.

## Discussion

The European honey bee gut has a well-defined “core” microbiome that contains bacteria from only a few (i.e., less than 10) phylotypes [44, 45]. According to metagenomic studies *Gilliamella*, *Snodgrassella*, *and Lactobacillus* ssp. were the most frequently found species in worker bees; *Bifidobacterium*, *Frischella*, *Bartonella*, *Parasaccharibacter*, and *Gluconobacter* were less frequently found, but these genera are still regarded as part of the “core” microbiome [17, 21–25, 44, 45]. These mostly anaerobic, facultative anaerobic or microaerophilic bacteria have co-evolved with the honey bee host [44], and their presence is considered to be important for honey bee health [46]. The bacteria in the honey bee gut play important roles in digestion (e.g., carbohydrate utilization), nutrition, weight gain, preservation of pollen, disease resistance, pheromone production, reproduction, endocrine signaling, immune function, and honey production [17–19, 47]. Furthermore, gut bacteria have the ability to inhibit pathogens, such as the chalkbrood fungus [20]. Like bacteria in the human intestine, most bee bacteria are found in the distal part of the gut where most occupy specific niches [17]. Bees exchange gut bacteria with other bees of the same colony through social interactions [28, 48]. It is likely that, as with humans, changes to the bee gut microbiota can lead to a reduced host fitness [49].

The gut microbiota in bees from Australia has not been extensively tested, with a notable exception of a recent study that investigated the microbiome composition in European honey bees and stingless bees (tribe Meliponini) from Queensland [45]. We isolated and characterized aerobic gut bacteria from European honey bees (nurse bees) that were collected from healthy hives, chalkbrood-infested hives and from hives that had recently recovered from chalkbrood. We also surveyed honey bees that were especially bred for enhanced hygienic behavior [41]. In the Australian late spring, summer and early autumn, bees from healthy colonies had 10^7^–10^8^ aerobic bacteria per g of bee gut (counted using TSA, a general purpose, non-selective medium for the cultivation and isolation of fastidious and non-fastidious bacteria; TSA has been noted as useful for this purpose [50]). Colonies with signs of the chalkbrood disease (from the same apiary) had significantly fewer aerobic bee gut bacteria (10^4^–10^5^ bacteria per g) than bees from healthy colonies. We also followed bacterial numbers in chalkbrood-diseased colonies over time, which revealed that bacterial numbers recovered when a colony no longer showed signs of chalkbrood. This observation establishes a direct correlation between the number of culturable aerobic bacteria in the gut of individual nurse bees and the health status of a colony. The decrease in aerobic gut bacterial numbers of bees from chalkbrood-infected colonies could be due to homeostatic imbalances caused by the presence of the chalkbrood fungus, i.e., metabolic changes due to chalkbrood infection may create conditions that no longer support large bacterial numbers and a diverse microbial flora. A critical factor could be the depletion of nutrients caused by the nutritional demands of growing chalkbrood mycelia [51]. Furthermore, the build-up of chalkbrood mycelia in the gut of infected nurse bees restricts the amount of space that is available for bacteria [52]. Our findings suggest that the number of aerobic gut bacteria can be used as a diagnostic tool to determine the relative health status of a colony, independent and potentially ahead of “classic” chalkbrood disease signs (such as cells with dead larvae, or mummified larvae in front of the hive; [53, 54]). Future studies will determine whether other fungal bee pathogens have a similar effect on aerobic gut bacterial numbers. An interesting candidate is *Nosema apis*, a microsporidian fungus that, until recently, was classified as a protozoan parasite [55, 56].

Nurse bees combat disease by removing infected larvae from the hive [54], a behavior that also promotes recovery from chalkbrood [57]. Bees from the West Australian “Better Bees” program are descendants from *Apis mellifera ligustica* breeding stocks [58] that were selectively bred for high honey production, good brood viability, lack of aggressiveness, low incidence of brood diseases, and high colony population size [41]. The “Better Bees” program has been somewhat successful in providing West Australian beekeepers with quality queens for improved honey production [59]. However, a subsequent report states that the hygienic behavior of such bee lines can vary and may not be a reliable character [60]. We found that bees from the “Better Bees” program had consistently significantly higher numbers of aerobic bee gut bacteria compared with “normal” bees from apiaries in the same area. It is tempting to speculate that “Better Bees” possess an enhanced metabolic rate because they have higher concentrations of carbon sources in their gut, which allows for a greater number of gut bacteria. In this study, we did not look at the role of hygienic behavior in disease resistance. It would be interesting to determine aerobic bee gut bacterial numbers in other bee lines which have genetically distinct queens [61].

Our preliminary genetic characterization of 19 Australian chalkbrood-inhibiting isolates from different Australian locations identified four species clusters, i.e., *Bacillus* ssp. (cluster I), enteric strains (cluster II), *Macrococcus* sp. (cluster III), and *Frigoribacterium* sp. (cluster IV). *Bacillus* ssp. were the most frequently isolated bacteria in this study; we found *Bacillus cereus*, *Bacillus senegalensis*, *Bacillus pumilus*, *Bacillus flexus*, *Bacillus megaterium*, *Bacillus thuringiensis*, and *Bacillus macroides* in the honey bee gut. *Bacillus* ssp. and enteric bacteria (*Enterobacter aerogenes*) have previously been isolated from worker bees in the United States [50, 62, 63]. The isolation of *Bacillus cereus* should be of particular interest for the bee industry, because a strain of this species that was previously isolated from honey bees inhibits *Paenibacillus larvae*, the etiologic agent of European foul brood [64]. The isolation of *Enterobacter aerogenes* in Australian honey bees confirms previous reports of *E*. *aerogenes* in the bee gut flora of foraging bees from Tucson, Arizona [62, 63]. Interestingly, our cluster III and IV isolates (*Macrococcus hajekii* and *Frigoribacterium* sp.) have not previously been found in bees. Whether these bacteria represent a uniquely Australian contribution to the microbiome of honey bees remains to be seen.

This study did not aim at isolating the mostly anaerobic or microaerophilic “core” microbiome bacteria. Instead, we focused on facultative anaerobes and aerobes, the so-called “environmental” gut bacteria. We show that these bacteria occur in high numbers (i.e., 10^7^–10^8^ CFUs per gram of bee gut) during the foraging season for honey bees. This observation is based on the analysis of more than 400 bees from nearly 100 colonies that were sampled all around Australia, representing different climate zones and pollen/nectar sources. The majority of these “environmental” bacteria is brought into the hive by foraging worker bees who pass them on to nurse bees trough food and social interactions [65].

Gluconic acid is a simple sugar with anti-fungal properties that can be utilized for the biocontrol of the take-all wheat pathogen [33]. Here, we report that the gluconic acid producing *Pseudomonas* strain AN5 inhibits the chalkbrood fungus. By using transposon independent mutants that do not produce gluconic acid, we show that the production of gluconic acid is essential for the antifungal activity of strain AN5. We further demonstrate that *Pseudomonas* strain AN5 extracts act on the chalkbrood fungus through hyphal lysis and cytoplasmic leakage. Our observation that *Maccrococcus hajekii*, *Frigoribacterium* sp., and *Bacillus senegalensis* isolates also produced gluconic acid suggests a similar *modus operandi*. However, these strains produced less gluconic acid than *Pseudomonas* strain AN5 but exhibited a similar degree of anti-fungal activity. Therefore, gluconic acid may not be the only metabolite by which bee gut isolates inhibit the chalkbrood pathogen. Interestingly, gluconic acid is considered to be the honey component that is largely responsible for the anti-microbial properties of honey [66]. Bees produce gluconic acid in the hypopharyngeal glands and in the gut with the help of gut bacteria [67]; however, more research is needed to understand how much gut bacteria contribute to the total gluconic acid production. Furthermore, it will be interesting to determine if and to what extent the bacterial gluconic acid production contributes to the resistance against bee pathogens.

## Conclusion

In this study, we used culturable aerobic and facultative anaerobic honey bee bacteria as a simple tool to monitor colony health. The method allows the rapid analysis of many samples and results are available within two days. Our findings suggest that decreasing numbers of culturable aerobic gut bacteria in nurse bees are a prognostic marker for the outbreak of chalkbrood and that increasing numbers herald recovery. Therefore, counting bacteria could be developed as a rapid test for apiarists that allows to detect deteriorating colony health before other disease signs become apparent. Furthermore, our results suggest that chalkbrood-inhibiting bacteria from the bee gut could be used to develop a probiotic treatment for chalkbrood and potentially other fungal bee diseases.

## Supporting information

S1 Fig Flowchart for the isolation and characterization of aerobic gut bacteria in European honey bees (*Apis millifera*) from Australia. (TIF)

S2 Fig “Environmental” bacteria from the gut of a healthy European honey nurse bee (*Apis millifera*) from Australia. The gut of the bee (without the crop) was homogenized in 1 ml of nutrient broth, diluted, and aliquots of the homogenate was plated on (**A**) tryptic soy agar (TSA), (**B**) eosin methylene blue agar (EMB), (**C**) glucose calcium carbonate media (G-CaCO_3_), and (**D**) modified Gould S1 media (mS1 Gould). The photos show microbial colonies after 2 days of incubation at 25°C under aerobic conditions.
(TIF)

S3 Fig Detection of acid production by bromocresol purple. “Environmental” bee gut isolates 15H12Ew1.1 (**A**) and 21H2Ew2.7 (**B**) were grown on potato dextrose agar (PDA) supplemented with bromocresol purple (15 mg/L). Plates were incubated at 25°C for 2 days under aerobic conditions. Acid production is indicated by a color change from purple (pH > 6.8) to yellow (pH < 5.2).
(TIF)

S4 Fig Detection of gluconic acid by mass spectrum analysis. (**A**) A hydrophilic extract of the nurse bee gut isolate ET2 was analyzed using a VG QUATTRO II mass spectrometer (with the arrow indicating the 195 m/z peak characteristic of gluconic acid). (**B**) Results of collision-induced dissociation (CID) analysis of the 195 m/z peak in panel B, confirms the presence of gluconic acid polymers.
(TIF)
