# Supplementary figures and images for: Environmental gut bacteria in European honey bees (Apis mellifera) from Australia and their relationship to the chalkbrood disease
Source: PLoS One. 2020 Aug 28;15(8):e0238252. doi: 10.1371/journal.pone.0238252 (PMC7455043; doi:10.1371/journal.pone.0238252)

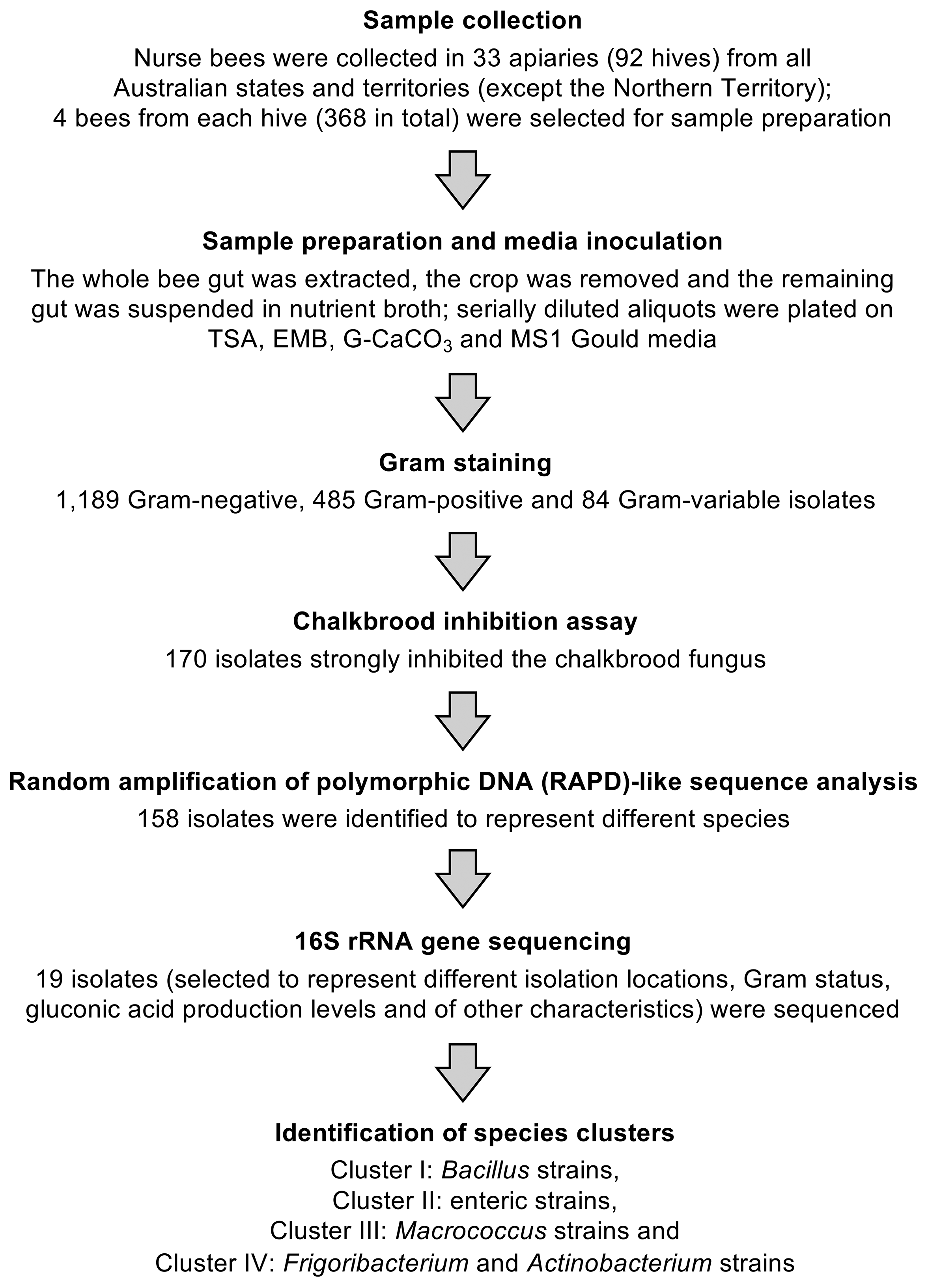

Supplement: S1 Fig — (TIF) [file pone.0238252.s001.tif]

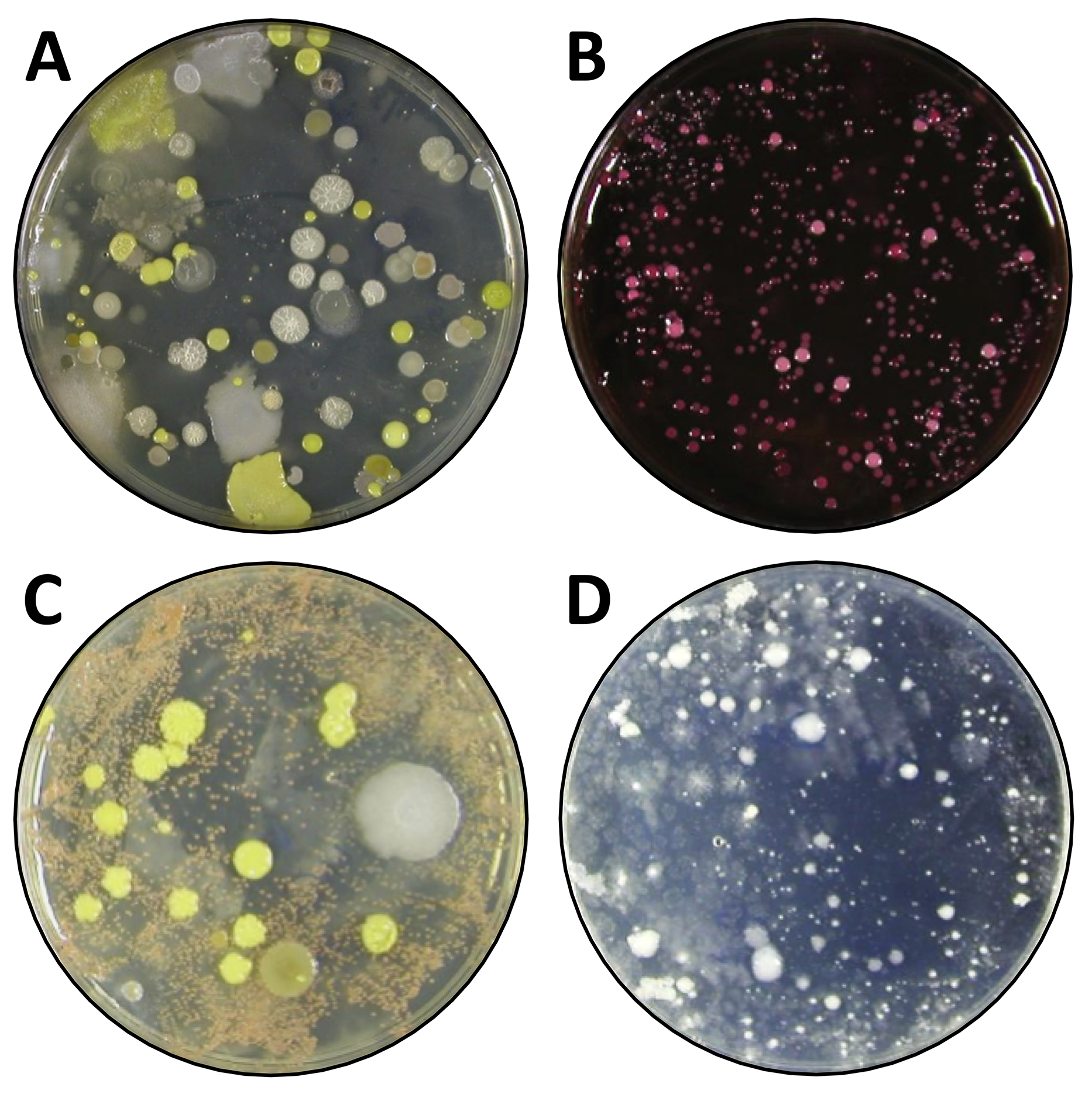

Supplement: S2 Fig — The gut of the bee (without the crop) was homogenized in 1 ml of nutrient broth, diluted, and aliquots of the homogenate was plated on (A) tryptic soy agar (TSA), (B) eosin methylene blue agar (EMB), (C) glucose calcium carbonate media (G-CaCO3), and (D) modified Gould S1 media (mS1 Gould). The photos show microbial colonies after 2 days of incubation at 25°C under aerobic conditions. (TIF) [file pone.0238252.s002.tif]

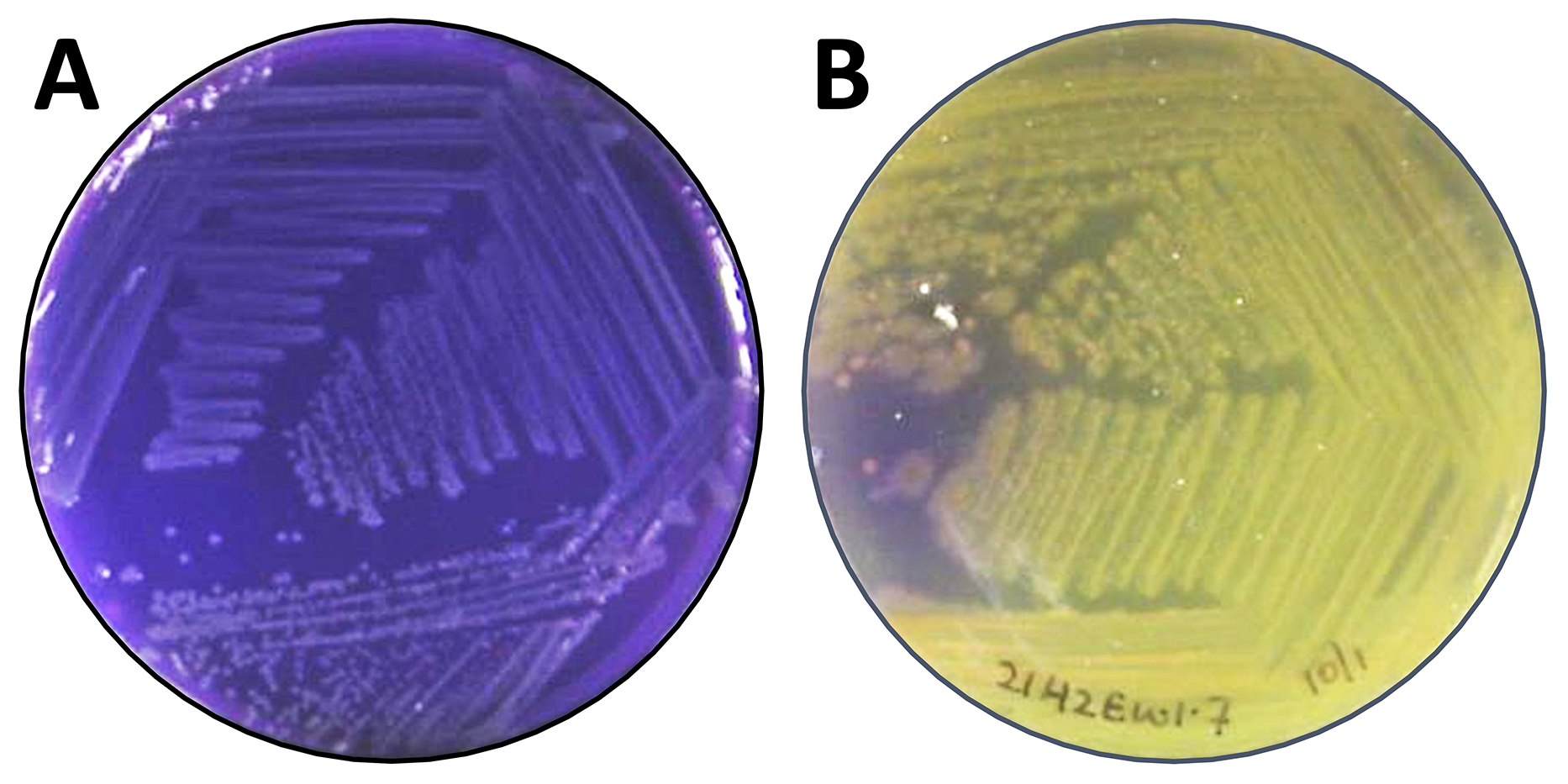

Supplement: S3 Fig — “Environmental” bee gut isolates 15H12Ew1.1 (A) and 21H2Ew2.7 (B) were grown on potato dextrose agar (PDA) supplemented with bromocresol purple (15 mg/L). Plates were incubated at 25°C for 2 days under aerobic conditions. Acid production is indicated by a color change from purple (pH > 6.8) to yellow (pH < 5.2). (TIF) [file pone.0238252.s003.tif]

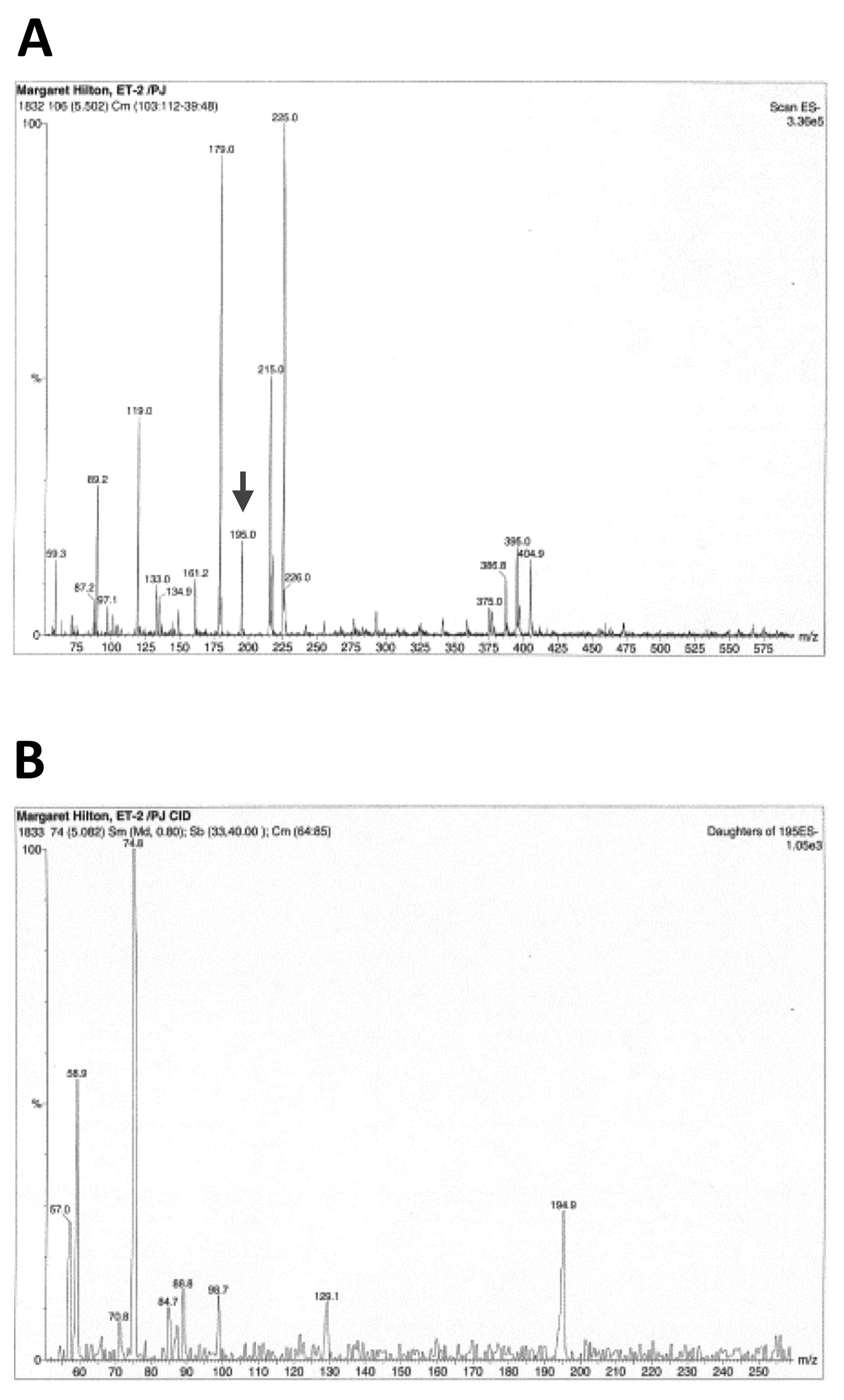

Supplement: S4 Fig — (A) A hydrophilic extract of the nurse bee gut isolate ET2 was analyzed using a VG QUATTRO II mass spectrometer (with the arrow indicating the 195 m/z peak characteristic of gluconic acid). (B) Results of collision-induced dissociation (CID) analysis of the 195 m/z peak in panel B, confirms the presence of gluconic acid polymers. (TIF) [file pone.0238252.s004.tif]
